# Gynæcological and Obstetrical Society of Baltimore

**Published:** 1889-06

**Authors:** 


### THE GYNÆCOLOGICAL AND OBSTETRICAL SOCIETY OF BALTIMORE.

*Stated Meeting, March 12, 1889.*

Dr. T. A. Ashby read an article entitled

THE ANIMAL SUTURE IN INTRA-VAGINAL PLASTIC SURGERY, the essential points of which are given herewith.

The evolution of intra-vaginal plastic surgery from the domain of a crude and unskillful procedure to a plane of high art and dextrous manipulation is due to the establishment of two principles—the principle of the speculum and the principle of the metallic suture, both elaborated by the genius of Marion Sims.

The principle of the metallic suture, which solved for the speculum the important idea of its vast utility in plastic vaginal surgery, was evolved by the genius of Sims as a necessary complement to the idea of successful exploration. His genius recognized in silver the necessary metal, and without knowledge of precedent in this direction he made successful application of his discovery. Plastic vaginal surgery at once became in the hands of Sims an assured fact, and its evolution has been the natural development of the idea originated by this remarkable man. The metallic suture had been used prior to the time of Sims by old Dr. Mettauer, of Virginia, who is credited with having used the iron wire suture as far back as 1830.

Silver wire possesses many advantages for plastic surgery. It has as a suture one disadvantage, but this is so striking that it is a barrier which must forever impair its otherwise striking advantages. The silver wire is non-absorbable and must, therefore, in every instance be removed when employed in plastic work. The wire suture exercises a cutting force and is easily imbedded by the swollen and inflamed tissues. Its removal must often be undertaken under difficulties.

In plastic vaginal work the mechanical presence of the wire is annoying to the patient and its removal is attended with greater or less pain.

For two years past he has almost entirely discarded the silver wire suture in operating upon the cervix and perineum, and with a growing experience is inclined to throw it overboard altogether. He was at first distrustful of the animal suture and alternated each suture with silver thread; he next used the animal suture all along the line of incision, save at the points of greatest tension, where he used the wire. Latterly he has trusted the animal suture altogether in many cases and finds the results far more satisfactory than he had dared hope. In no case has he failed to get good union, and in the majority of instances the line of union has been simply perfect. This has been especially true in operations upon the cervix.

In the double operation of trachelorrhaphy and perineorrhaphy, he performed the operation first named and then after a month or six weeks' interval closed the perineum. Within the past two or three years he has been able to abandon this plan and he now invariably does both operations during the same anæsthesia. The use of the animal suture first encouraged this plan. After closing the cervix with cat-gut the perineum is closed with the same material. The patient is placed in bed and kept upon her back for a week or ten days. At the end of this time repair has taken place, the suture is absorbed or dissolved, suture points closed and nothing further is required beyond a few days' convalescence. After all inflammatory swelling and soreness have disappeared the case is dismissed with the simple injunction to use the hot vaginal douche for several weeks.

To illustrate the advantages of the animal suture he selected from a number of cases brief histories of three cases illustrating the different procedures in which the suture was used.

TRACHELORRHAPHY.

CASE I. Mrs. D., aged 28, bilateral laceration of cervix of over four years standing. Lips somewhat hypertrophied, widely gaping and edges eroded. Mucous membrane secreting profuse muco-purulent discharge, constant pains in pelvis and back, nervous depression and general lowering of health.

The operation clearly indicated. Under chloroform anæsthesia, the lips of the wound were carefully pared, and closed with No. IV. carbolized cat-gut suture, manufactured by the Lister Manufacturing Company. Patient was placed in bed and at end of 7th day union was found complete, sutures absorbed and patient in comfortable condition in every way. On the 10th day the patient was up and going around her room. Her subsequent condition was entirely satisfactory.

TRACHELORRHAPHY AND PERINEORRHAPHY.

CASE II. Mrs H., aged 30, married, cervix badly lacerated on left side as high up as vaginal junction, lips of wound somewhat everted and hypertrophied, edges rough, granular, profusely secreting mucus and pus; patient bed-ridden since confinement nine months previous, general health greatly depreciated and nervous system broken down.

Perineum torn back to rectal septum during previous labors. The anterior and posterior vaginal walls relaxed and projecting through vulvar opening when in the erect position or during straining from coughing or vomiting. Pelvic circulation disturbed and sluggish, the vaginal tissues having a dark venous hue. The indications for the operation were as pronounced as one could find; under chloroform anæthesia, the cervix was repaired with carbolized cat-gut suture, sizes No. I and No. IV being used. The perineum was next denuded and closed with the same suture; at the end of eight days union was complete along the entire perineal line of suture; no attempt was made to ascertain condition of cervix at this time. Hot water injections were employed twice daily after 5th day. Upon examination of cervix on 14th day union was found satisfactory. The suture had disappeared by disintegration, only filaments being found in the vagina. The subsequent progress of the case has been good, the patient being able to resume her domestic duties and continues to improve in health.

RUPTURE OF PERINEUM THROUGH RECTAL SEPTUM.

CASE III. Mrs. McC., aged between 30 and 35 years, married, gave birth to second child with great difficulty; infant 5 months old at time of operation. Physician in attendance at time of delivery failed to repair the tear, which extended 1½ inches up the rectal septum and through entire perineum. The condition of patient soon become extremely distressing. She had lost all control of her lower bowel and was being daily reduced by diarrhœal discharges. I was invited to see the patient in consultation and then requested to operate.

Under chloroform anæsthesia the parts were carefully denuded and then closed with carbolized cat-gut, sizes Nos. I and

IV being used. The line of union was found complete at end of 8th day, with single exception of one point at upper and inner edges of the vaginal wound where slight sloughing occurred. A small opening not larger than a quill remained unclosed. After evacuation of bowels and use of hot water for several days, closure at this point took place. Subsequent results have been satisfactory, the patient having been restored to health and comfort and to usefulness in her family.

Claim is made for the superior excellence of the animal suture upon two points: First, it is non-irritating; second, it is self-removable. If it can be shown to have all the other good qualities of the silver wire and those additional advantages it becomes *par excellence* the suture for plastic work. The procedure necessary for the removal of the silver suture after operations within the vagina or upon the perineum is in many cases almost as troublesome as that required for placing them. There are few patients who do not complain bitterly of the pain induced by the attempt to remove the suture, and many will not assent to its removal without an anæsthetic. Many patients as they lie in bed and think over the pain and distress attending the removal of the wire suture, anticipate this work upon the part of the operator with holy horror. He has had more complaints from patients from this source than from the dread of the primary operation.

In simple perineal operations the wire suture is disagreeable and often painful from its mechanical presence during the entire time it remains *in situ.* Its removal is always attended with pain unless an anæsthetic is employed. Local anæsthesia with cocaine has not been sufficient in his experience to overcome this pain. Silver wire does not yield to pressure to the same extent as the animal or silk suture, and changes in the positions of the body are much interfered with in consequence, when it is used. This is to some extent an objectionable feature. The greater the amount of motion allowed a patient within certain limits, the greater the comfort of the horizontal position. After using the cat-gut suture, he ties the patient's limbs together and allows her to roll from side to side at will. This lateral roll works no disadvantage and occasions no discomfort; it is a positive gain.

The following are the objections urged against the cat-gut or animal suture.

First. It is claimed that it is less easily adjusted than silver wire, that the edges of the flaps approximate with less neatness and ease of manipulation, that the knot is liable to slip or loosen in attempts to tie. These defects are admitted, but they can be entirely overcome with care and painstaking work. He has no difficulty in bringing the flaps in proper apposition or in holding them there until the suture is carefully tied or buttoned. This may be done by using a vulcellum forceps, a tenaculum or a temporary wire suture. The knot will not slip or untie if it is looped properly. He uses the surgeon's loop and makes three ties. The ends of the suture are left from $1\frac{1}{2}$ to 2 inches long. To prevent the knots from untying, he covers the suture and wound with iodoform, which, in addition to its healing and antiseptic properties, dries the suture and securely holds the knot.

Second. It has been claimed that the cat-gut suture is not strong enough for cervical and perineal operations, and that it disintegrated before primary union could take place. His experience disproves this assertion. He finds the suture is sufficiently strong for this work if it is carefully selected and tested before being used. The best results will be secured by using sutures of different sizes. He varies the size according to the amount of tension or strain imposed on the suture. In very fine plastic work a very fine suture should be used and *vice versa.* A properly prepared cat-gut suture will hold *in situ* from 4 to 14 days. Primary union will take place within 48 to 72 hours in the majority of cases of cervical and perineal operations, so that the self-removable suture will prove sufficient for these procedures.

In conclusion he offered the following summary:

1st. The cat-gut suture properly prepared and selected will be found sufficiently strong and durable for operations upon the cervix and perineum in the vast majority of cases.

2d. With careful manipulation it can be placed *in situ* with sufficient neatness and fitness to secure perfect union of the denuded surfaces by primary intention.

3d. It is self-removable, and therefore does away with the necessity of further interference after union has been secured.

4th. It gives no distress while union is in progress.

5th. It makes the operation of trachelorrhaphy and perineorrhaphy during the same anæthesia a very simple procedure.

DISCUSSION.

Dr. W. E. Mosley had tried various forms of sutures and his conclusions were as follows: Taking cervix operations as a whole, the metallic was far preferable to the animal suture. The only class of cases where the animal suture was permissible was when the lips were thin and easily brought together. When any amount of cicatricial tissue had to be removed animal sutures could not be depended upon, as the tension hastened absorption, and even before any absorption had taken place they would stretch, so letting the denuded surface separate. In this respect silk-worm gut differs from the animal suture used. The edges of the denuded surface can be more perfectly adapted to each other with the metallic suture and the degree of tension more exactly estimated. He did not approve of tying animal sutures when used in cervix operations but thought they should be shotted, as by this means less traction on the ligaments is necessary, and the tension of the suture more readily regulated. Another advantage the metallic suture has is that it draws the denuded surfaces more smoothly into apposition while all animal sutures act like a draw string at the mouth of a purse.

In operations upon the anterior and posterior walls of the vagina, cat-gut sutures are often very serviceable and much more comfortable for the patient. In fistulæ, the supporting, splint-like action of the metallic suture is often an advantage. In Dudley's perineum operation the suture is continuous and an animal tissue must be used. In Emmett's operation, although animal sutures give good results, the central suture forming the fourchette had better be of metal.

Of the animal sutures, cat-gut is the most universally applicable, and that which has been chromicized and then kept in Juniper oil the most reliable. Silk-worm gut is stiff and has a thin cutting edge, and unless pretty thoroughly softened in hot water is liable to split when being tied.

Of the metallic sutures, pure carefully annealed silver wire is best.

In conclusion, he would not use animal sutures where there was much tension or when an exact coaptation of the denuded surfaces was necessary, as he believed silver wire would answer the purpose much better. If animal sutures were used in cervix operations, they should be shotted and not tied. In operations upon the vaginal wall, near the vulva, they can be readily tied.

Dr. H. P. C. Wilson said that he had always been so perfectly satisfied with his results with wire that he did not care to try new and unaccustomed materials. Seldom heard complaints on removal, and never gave an anæsthetic for this trivial operation.

Dr. T. A. Ashby said, in closing, that he began practice with a prejudice in favor of the metallic suture, but experience had taught him to prefer cat-gut. Within the last year he had used the wire in the cervix but twice. He admits that wire is sometimes the best in these operations, but not often, reviewed the remarks and explained just when wire might be best. The cat-gut should be tested thoroughly before introduction. When the cervix is high in vagina, he ties with catch forceps. Appearance of wound is not so nice as with wire, but result is as good.

Dr. Charles O'Donovan, Jr., then read an article entitled

IMPACTED RETROVERSION OF THE GRAVID UTERUS, WITH A CASE.

The following summary presents the salient points.

On August 12th, 1888, I was called hurriedly to see Mrs. F., aged 35, white, of Irish parentage, the mother of six children. I found her pale and thin, with an anxious countenance, looking so exhausted and exsanguine that I thought at first, for I had never seen her before, that I had a case of hæmorrhage of some kind to deal with. She told me that her family physician had been treating her during the past ten days for violent pelvic pains, that had been recurring now and again at irregular intervals during that time; these had gradually become less and less frequent, and were now altogether absent; but during the last three days a tumor had developed in her abdomen, and had grown rapidly, in spite of all that her physician could do.

In answer to my inquiries, I learned that she had missed three periods, but did not believe herself pregnant, though subsequently she proved to be so; she had had a slight flow six weeks ago, subsequent to over-exertion, but this passed off after a few hours, and she had paid very little attention to the occurrence. About a week ago she began to have pains in her pelvis, and lower abdomen, which seemed to be caused by some trouble situated in or about the bladder, for, as the pains became more violent, she noticed that a greater effort was required for micturition, and that each time fewer and fewer drops of urine could be expelled; four days ago she failed to urinate, notwithstanding the most violent efforts, accompanied by excruciating pains, and she had not since passed more than a few drops from her bladder. During this time she suffered intensely, but to-day she felt easier. Her bowels had acted fairly well, though she was somewhat constipated. Inspection of the abdomen revealed a tumor, reaching from the symphysis pubis to within two inches of the umbilicus, rising prominent from the belly, and bulging forward, even while she lay on her back. It presented a hard, elastic feeling, when touched, and gave a perfect wave of fluctuation, even when tapped as delicately as possible, rendering it certain that I had to deal with a tense cyst of some sort. As the woman was quite frail, and the abdominal walls were very thin, by causing her to flex her thighs on her body, thus relaxing completely the abdominal muscles, I could push my fingers around the tumor at the sides and above, clearly defining its globular mass. I next attempted to introduce my finger into her vagina, but encountered unexpected resistance; the vagina was almost obliterated by a tumor, occupying Douglas' cul-de-sac, which was large enough to fill the entire pelvis so completely that I could make out nothing more than I could feel by the vagina, except that the overcrowded condition of the pelvis was shown more clearly. Though I doubted her report about passing no urine for four days, she may have told the truth, as the weather was quite hot and her skin was acting freely; however I did not allow many minutes to pass before I had introduced a long rubber catheter into her bladder, through which slowly flowed more than two quarts of very dark urine, having a strong ammoniacal odor; during this process the abdominal tumor gradually collapsed, and she complained of a good deal of pain, though she felt much relieved at its completion. Having removed this obstruction, I expected to make a satisfactory vaginal examination, but was disappointed when I found matters very little improved; the only advance that I could make was that by bi-manual examination I could satisfy myself that the uterus was retroverted, but whether the mass in Douglas' cul-de-sac was the fundus alone, or the fundus dragged back by a fibroid, or the pregnant uterus, I could not distinguish. So, after ordering dessertspoonful doses of Castor oil, to be given at intervals of four hours, until her bowels had been freely moved, I left her resting quietly.

When I visited her the next day, I found that the oil had acted nicely, giving her several movements containing many hard lumps of fœcal matter, but she had passed no urine since I had seen her, so I again used the catheter, before examining her by the vagina. The condition of her pelvis had changed somewhat since the day before, the evacuation of her rectum having released the uterus so that it could be moved easily without pain, but I could not push the enlarged fundus through the ligaments into its proper position; after each effort it would fall back into the recto-vaginal pouch from which it had been lifted; it seemed to be about as large as a good sized orange, but contained no fibroids that I could distinguish. I instructed her sister in the use of the catheter, which she readily learned, and ordered that it be used twice a day, if she failed to relieve herself; also that she should use the hot vaginal douche each night and should keep her bowels moving regularly with Castor oil, and that half a gallon of warm water be injected into her bowel each night and morning; as she was very pale and anæmic, I directed Blancard's pills, one after each meal. After this I saw her every two or three days, finding her better each day, though compelled to use the catheter regularly, until September 1st, ten days after my first visit, when she told me that she found herself able that morning to pass her water without difficulty, and felt relieved also of a feeling of discomfort and weight in the pelvis. Upon examining her, I was surprised to find that the uterus had risen spontaneously out of the pelvis, and could be felt above the symphysis; the os remained rather high in the pelvis, but was easily reached, having a deep bilateral laceration. Her other pelvic organs seemed perfectly healthy.

The interest in this case arises from two points:

1st. The diagnosis of the nature of the tumor, and

2nd. The spontaneous return of the uterus to its proper position after two repeated failures of efforts made to replace it by the finger.

That this retroverted gravid uterus, after resisting efforts at reposition, as strong as I dared make them, for the dread that I might be dealing with such a condition was always present notwithstanding her assurance that she could not believe herself pregnant, should rise above the obstructing ligaments spontaneously after a few days of such simple treatment as rest in bed and vaginal and rectal injections of warm water twice daily, is a matter that merits consideration. I had attempted to replace it while she lay on her back, but failed; I tried next while she was in the genu-pectoral position, giving full time for the forward displacement of all the abdominal and pelvic viscera, and admitting atmospheric pressure by retracting the perineum; this alone having failed to push the uterus into its proper place, I assisted rather forcibly with two fingers in the vagina, and after the failure of this method, while the woman retained the same position, I tried unsuccessfully to accomplish my purpose by inserting my finger into the rectum hoping to gain a longer leverage. This occurrence, however, fully demonstrates the utility of warm injections, particularly into the rectum, for to that influence more than any other I am inclined to ascribe the favorable termination of the case, by bringing about relaxation of the ligaments that presented but a few days before an impassable barrier.

